# Supplementary material for: Glycerol-Induced Powdery Mildew Resistance in Wheat by Regulating Plant Fatty Acid Metabolism, Plant Hormones Cross-Talk, and Pathogenesis-Related Genes
Source: Int J Mol Sci. 2020 Jan 20;21(2):673. doi: 10.3390/ijms21020673 (PMC7013599; doi:10.3390/ijms21020673)
Supplement: Supplementary file 1 [file ijms-21-00673-s001.zip › supplementary files/Figure Supplement.pdf]

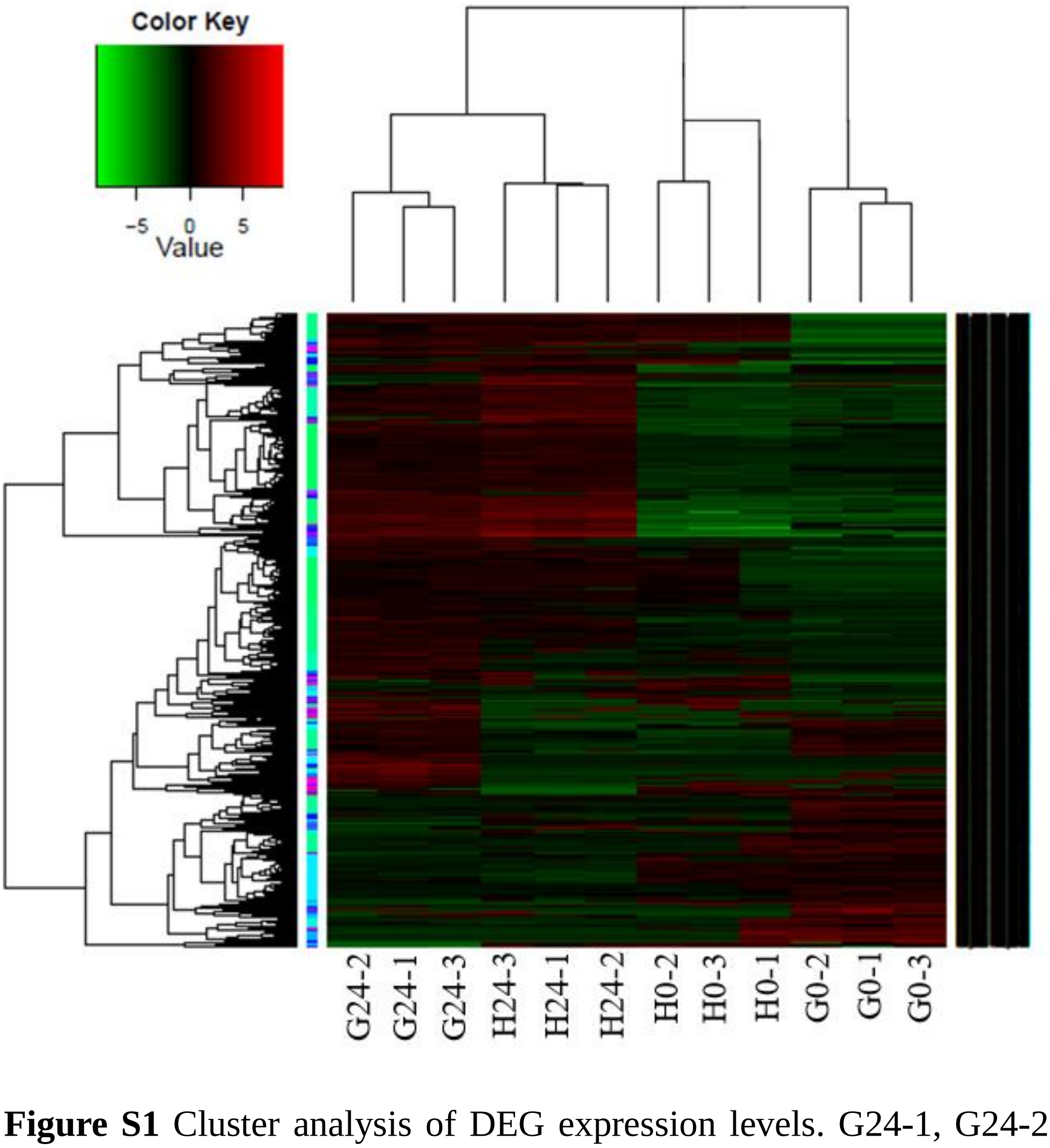

Figure S1 Cluster analysis of DEG expression levels. G24-1, G24-2, G24-3: three biological repetitions of G24 group; H24-1, H24-2, H24-3: three biological repetitions of H24 group; G0-1, G0-2, G0-3: three biological repetitions of G0 group; H0-1, H0-2, H0-3: three biological repetitions of H0 group; The color represents the expression level (absolute intensity), where red indicates a higher expression level and green indicates a lower expression level of DEGs.

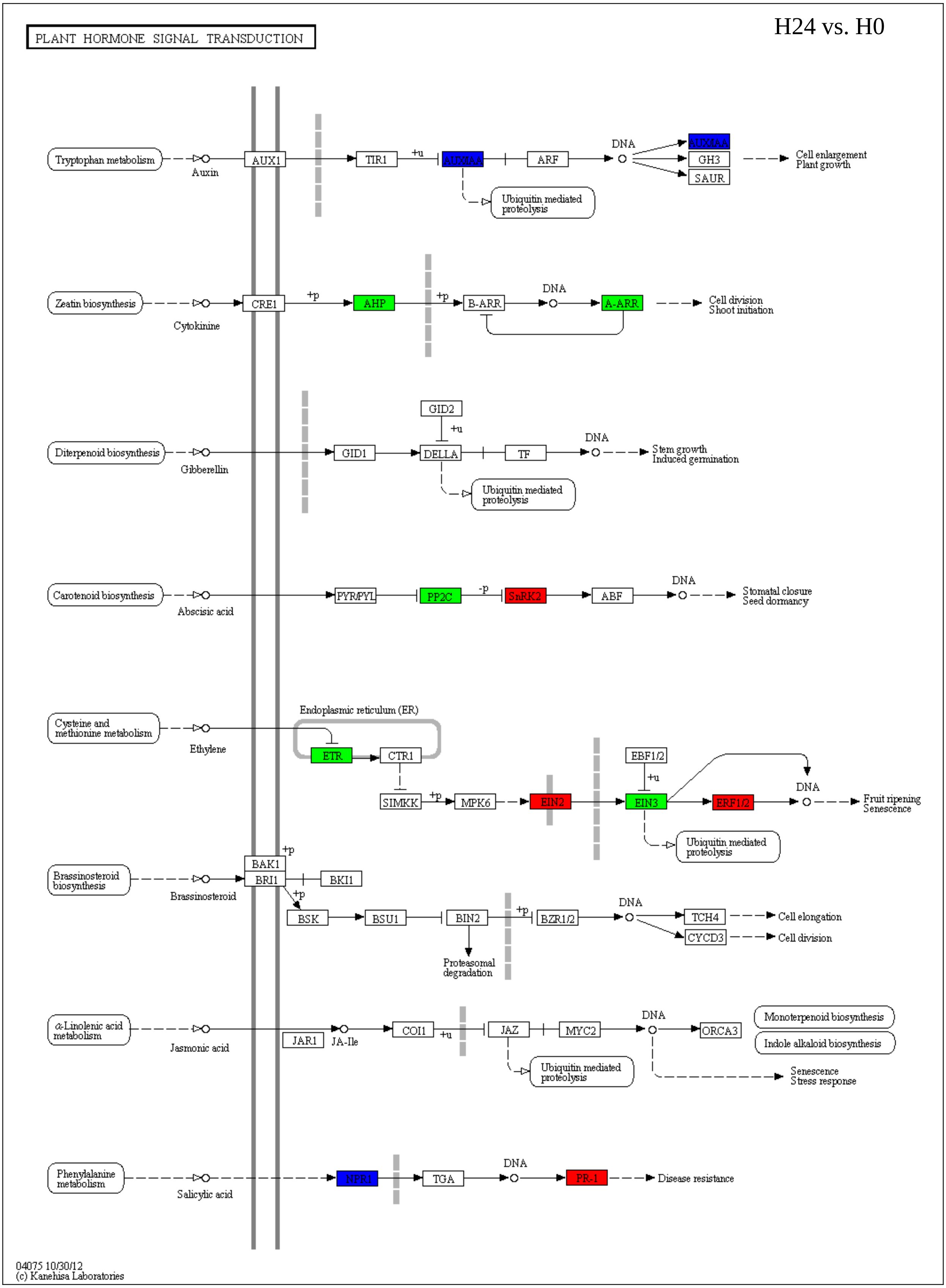

Figure S2 Gene expression in the plant hormone signal transduction pathway (Ko04075) changed in response to *Bgt*-infection. The red color indicates gene expression induction in H24 group; The green color indicates gene expression reduction in H24 group; The blue color indicates that expression patterns of some annotated genes were induced and some homologous genes were reduced in H24 group.

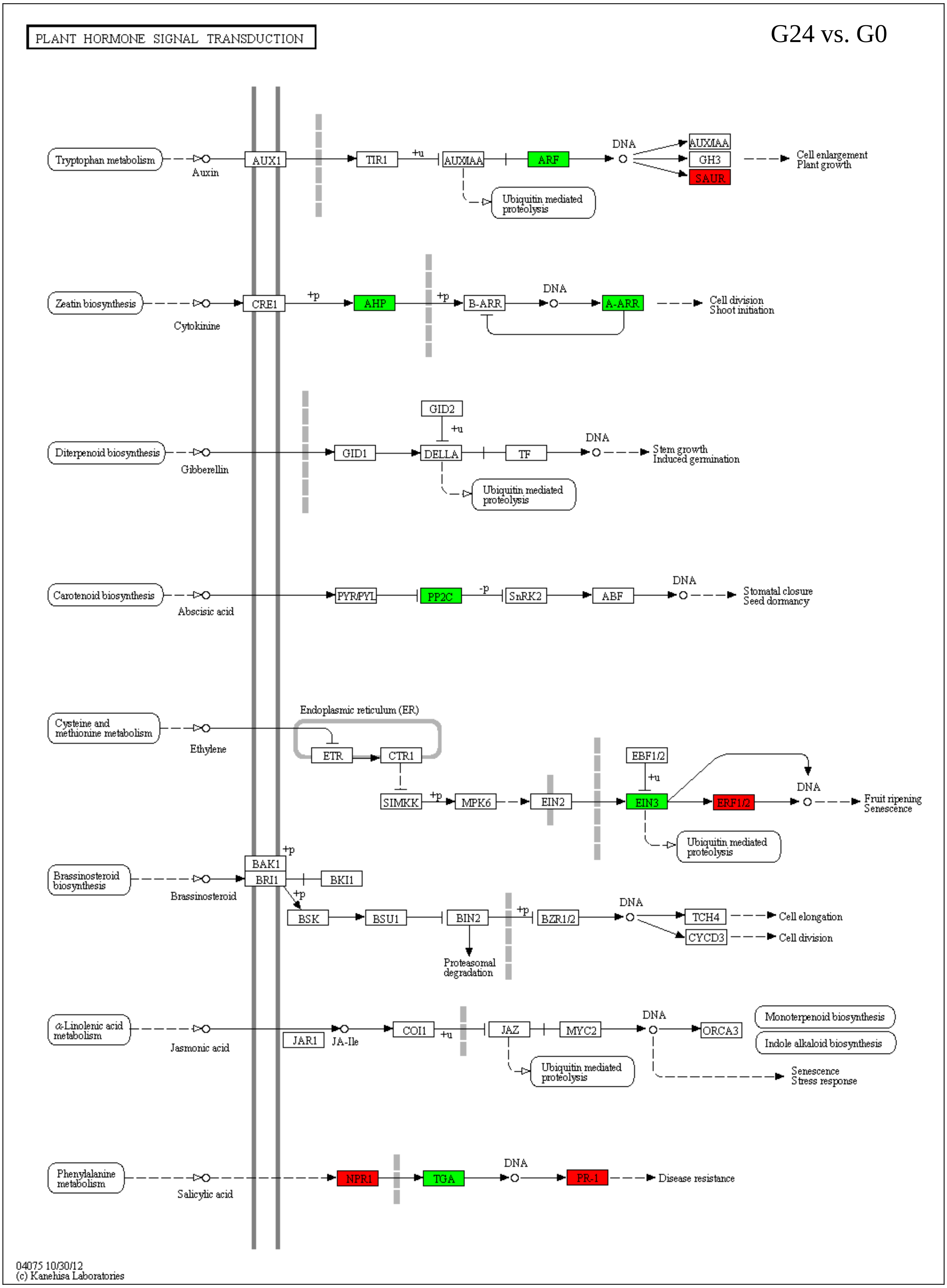

Figure S3 Gene expression in the plant hormone signal transduction pathway (Ko04075) in response to *Bgt*-infection. The red color indicates gene expression induction in G24 group; The green color indicates gene expression reduction in G24 group.

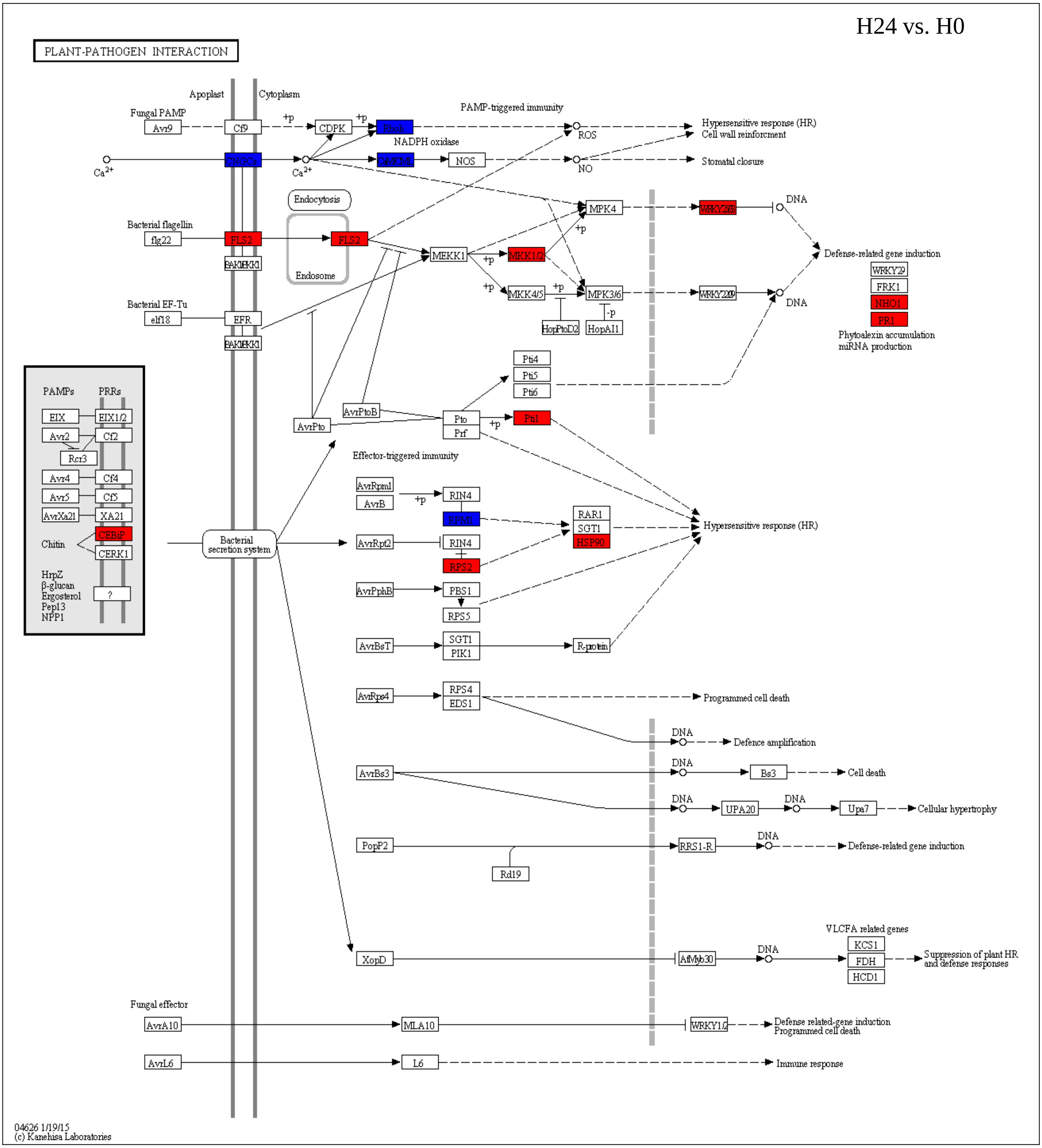

Figure S4 Gene expression in the plant-pathogen interaction pathway (Ko04626) changed in response to *Bgt*-infection in the water-treated plants. The red color indicates gene expression induction in H24 group; The green color indicates gene expression reduction in H24 group; The blue color indicates that the expression patterns of some annotated genes were induced and some homologous genes were reduced in H24 group.

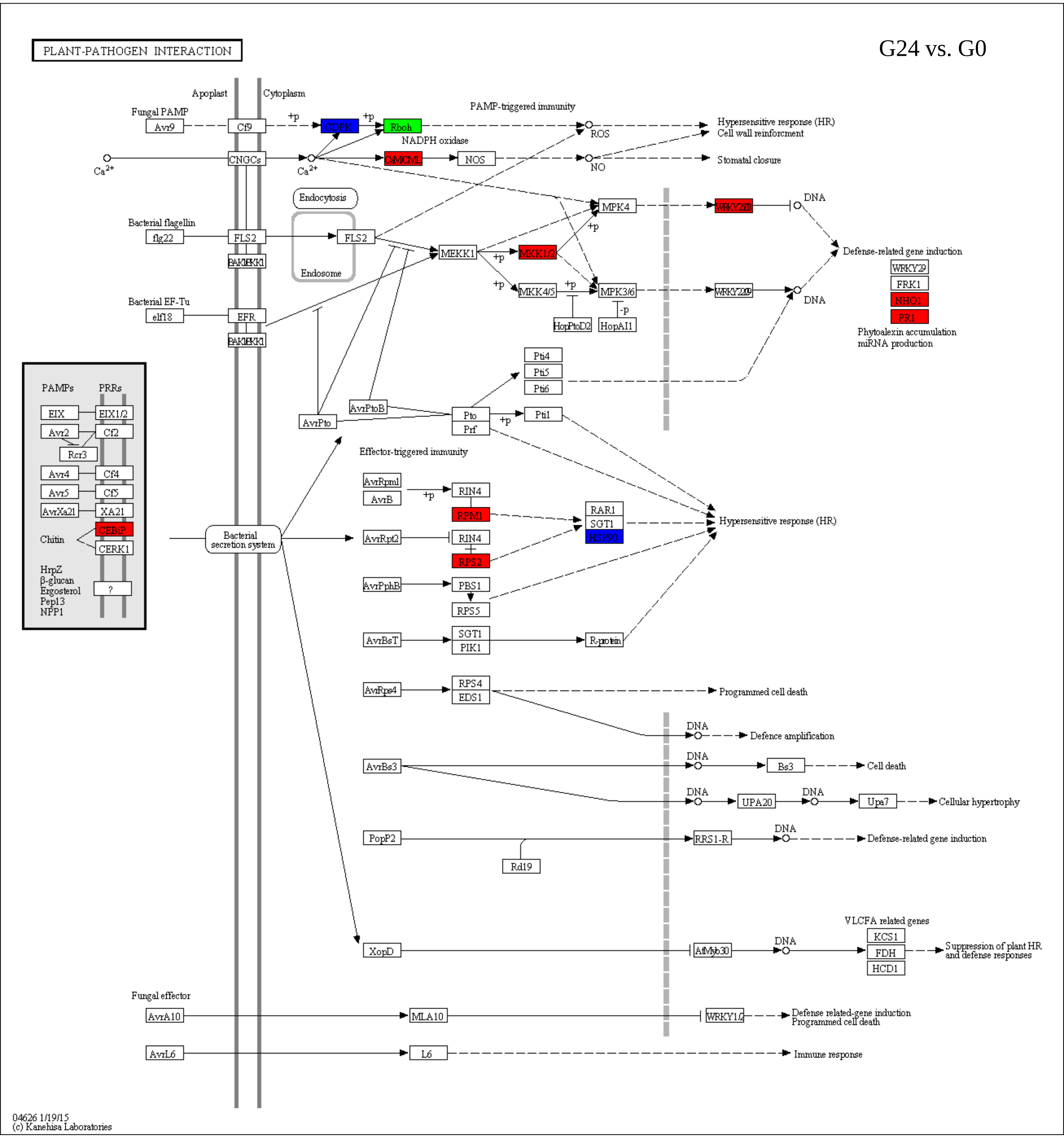

Figure S5 Gene expression in the plant-pathogen interaction pathway (Ko04626) changed in response to *Bgt*-infection in the glycerol-treated plants. The red color indicates gene expression induction in G24 group; The green color indicates gene expression reduction in G24 group; The blue color indicates that the expression patterns of some annotated genes were induced and some homologous genes were reduced in G24 group.

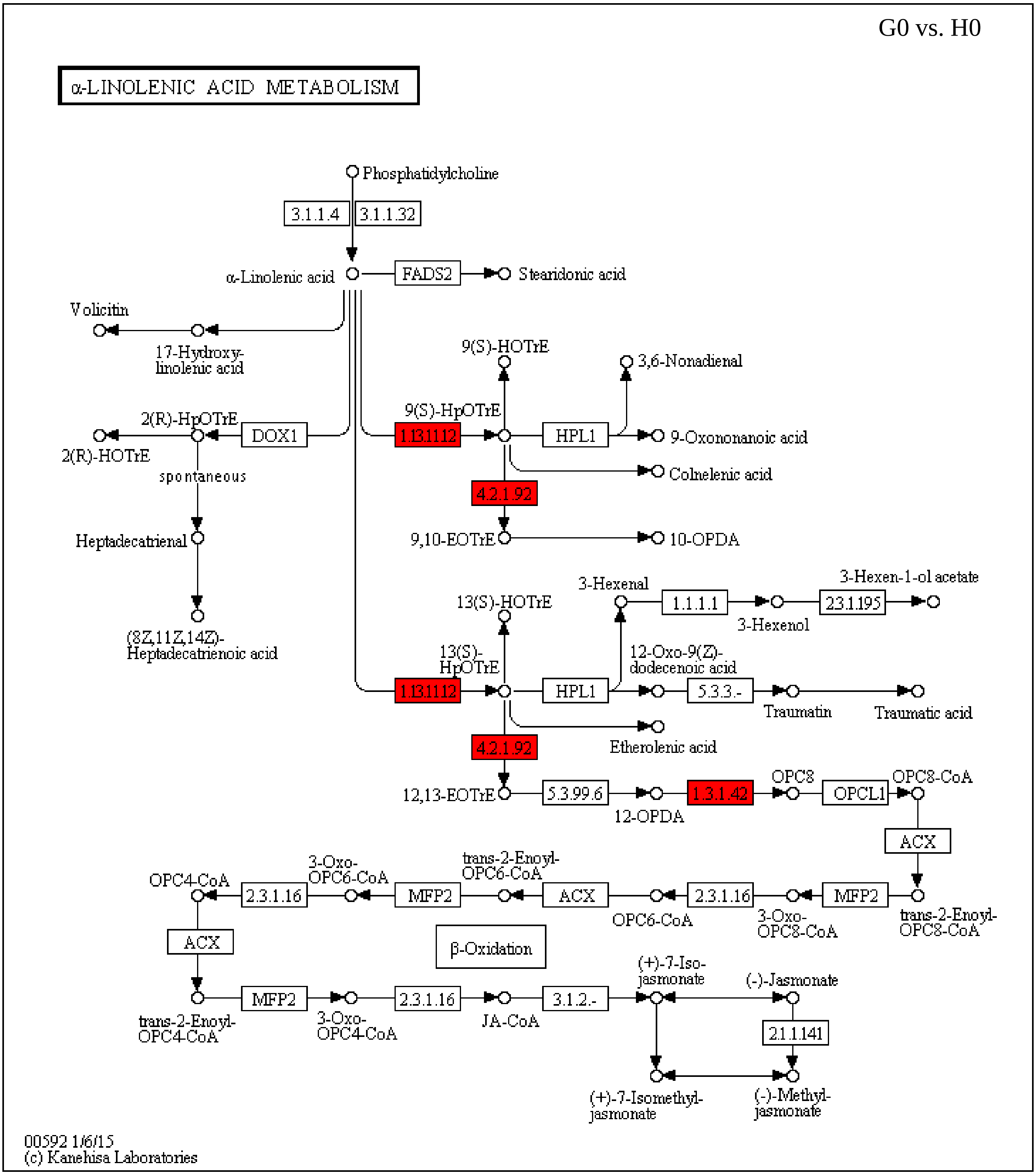

Figure S6 Gene expression in the a-linolenic acid metabolism pathway (Ko04626) in response to glycerol. The red color indicates gene expression induction in G0 group.

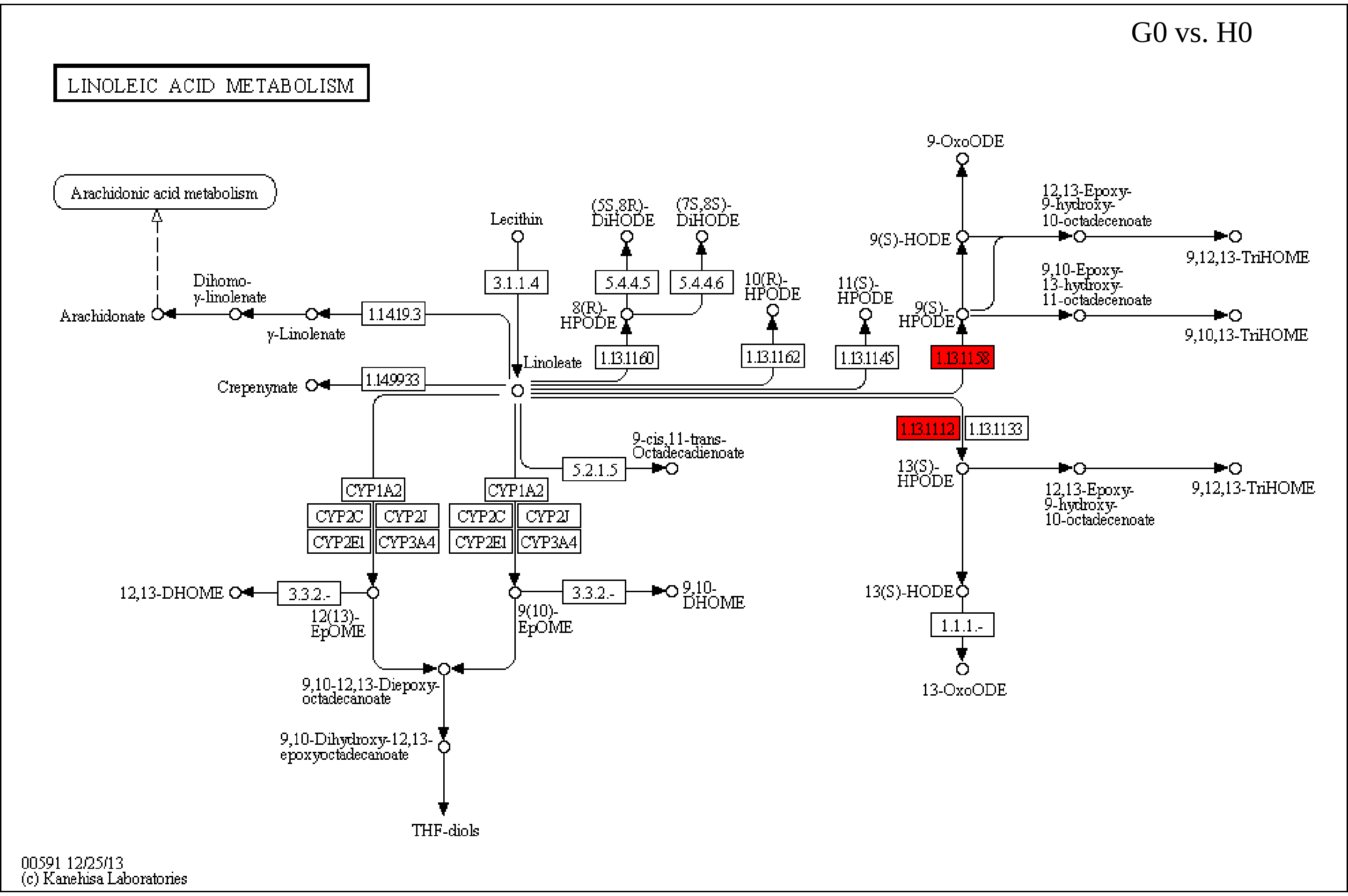

Figure S7 Gene expression in the linolenic acid metabolism pathway (Ko00591) in response to glycerol. The red color indicates gene expression induction in G0 group.

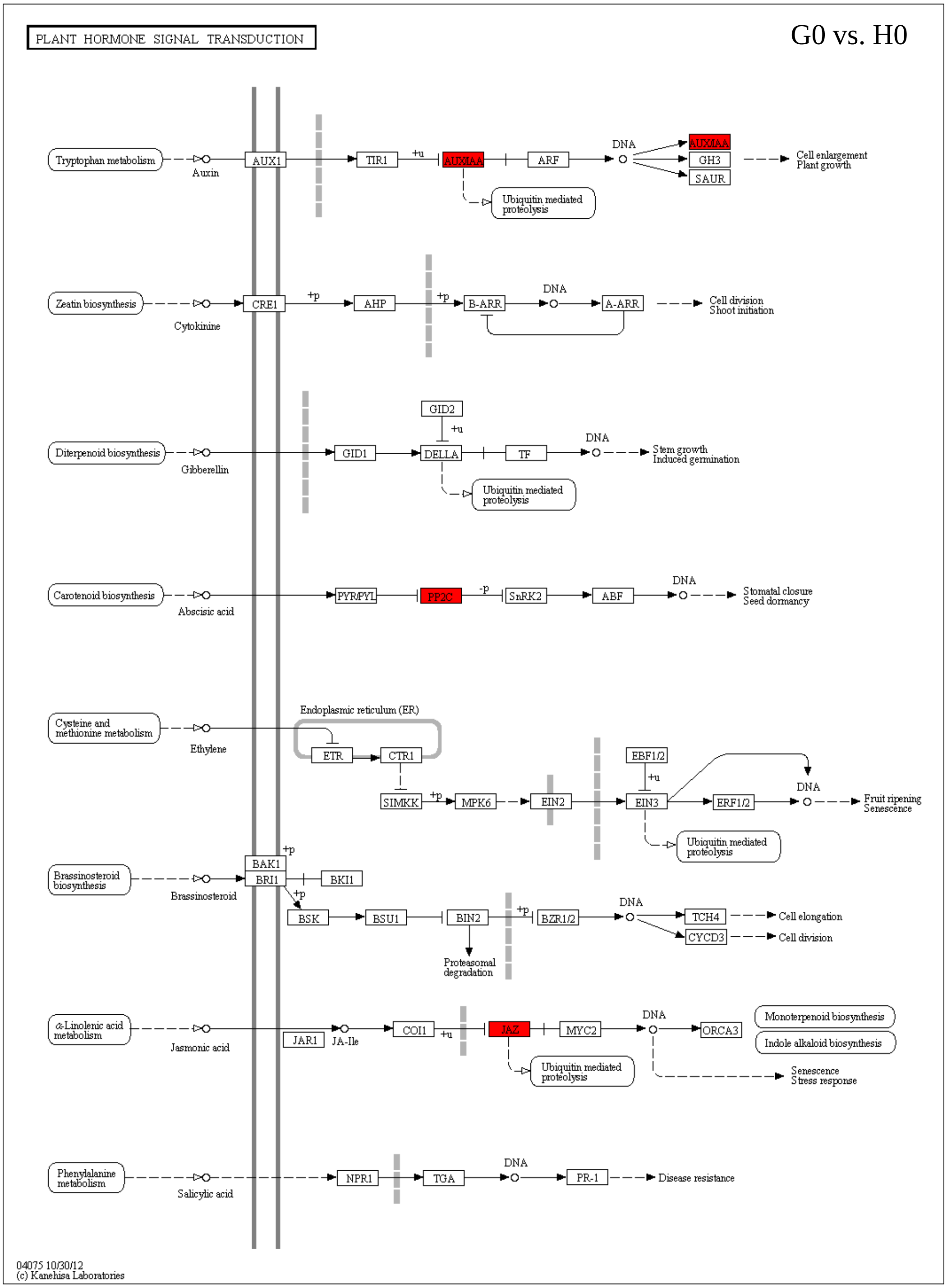

Figure S8 Gene expression in the plant hormone signal transduction pathway (Ko04075) in response to glycerol. The red color indicates gene expression induction in G0 group.

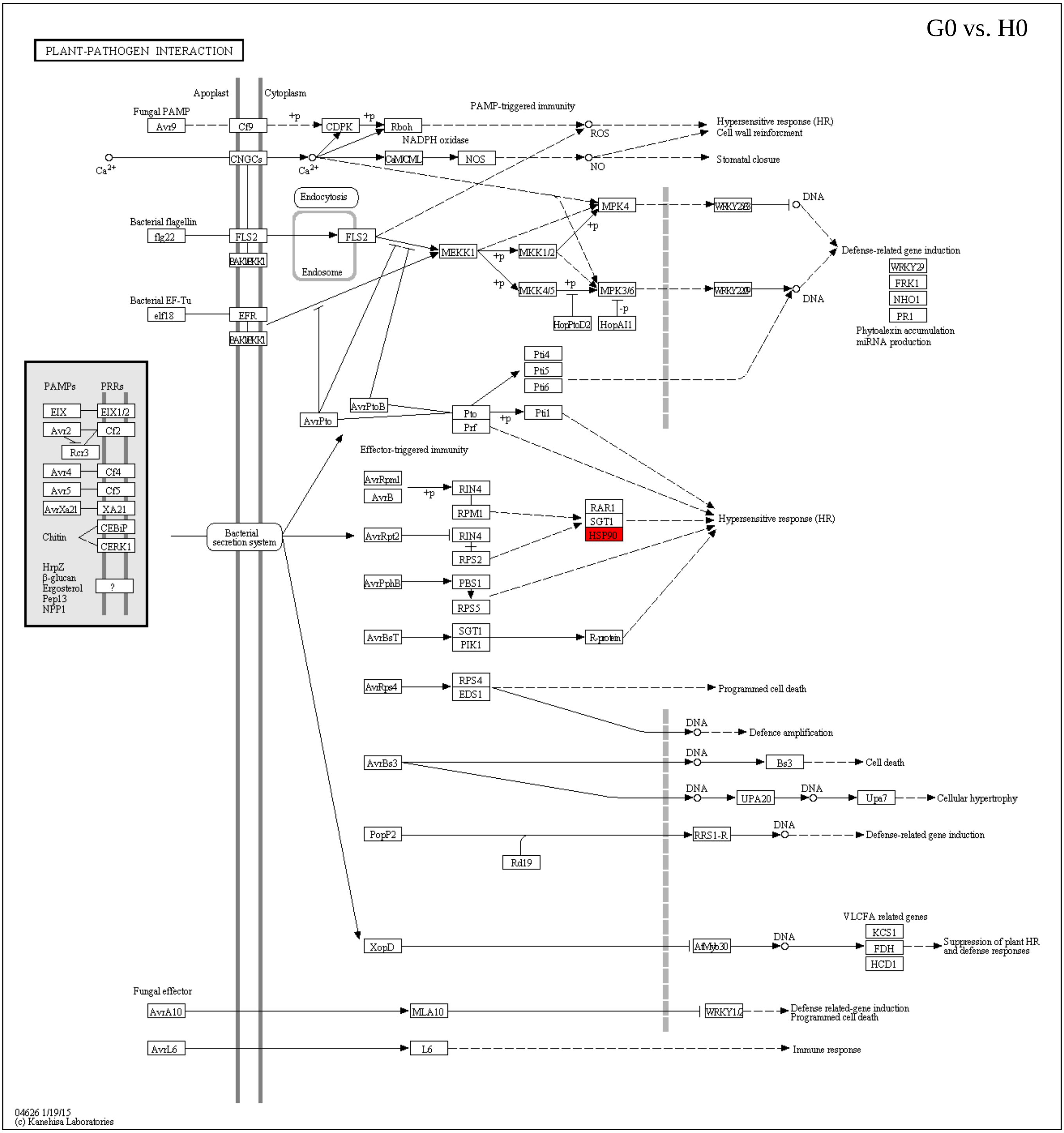

Figure S9 Gene expression in the plant-pathogen interaction pathway (Ko04626) changed in response to glycerol. The red color indicates gene expression induction in G0 group.

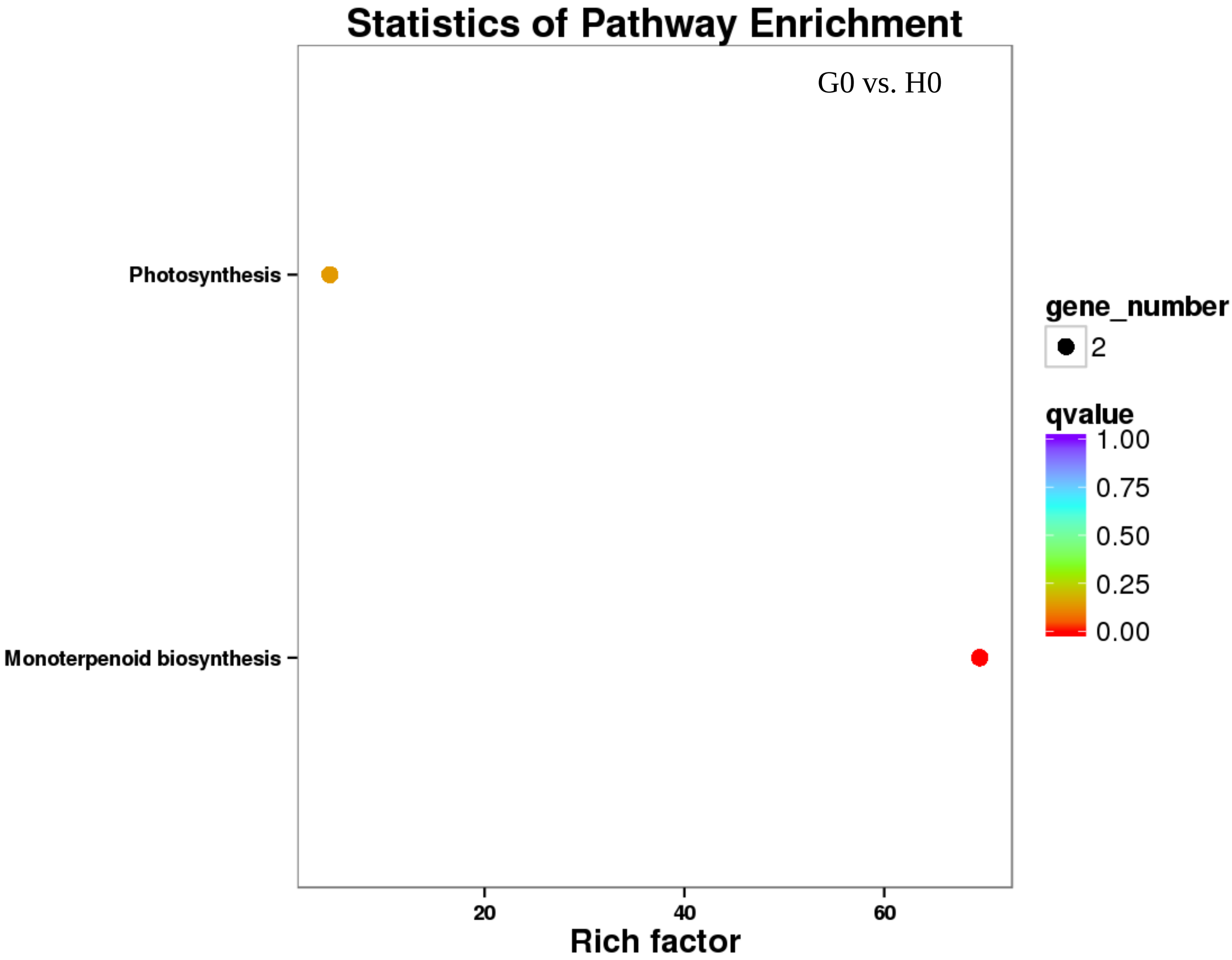

Figure S10 Enriched KEGG pathways of the down-regulated DEGs in response to glycerol application. The color represents the Q value as shown in the legend. Q values are the *P* values corrected for multiple hypothesis testing and range from 0 to 1. The closer the Q value is to zero, the more significant the enrichment. The horizontal axis indicates the rich factor, where higher a rich factor indicates a greater degree of enrichment. The size of each circle indicates the number of DEGs in that pathway.

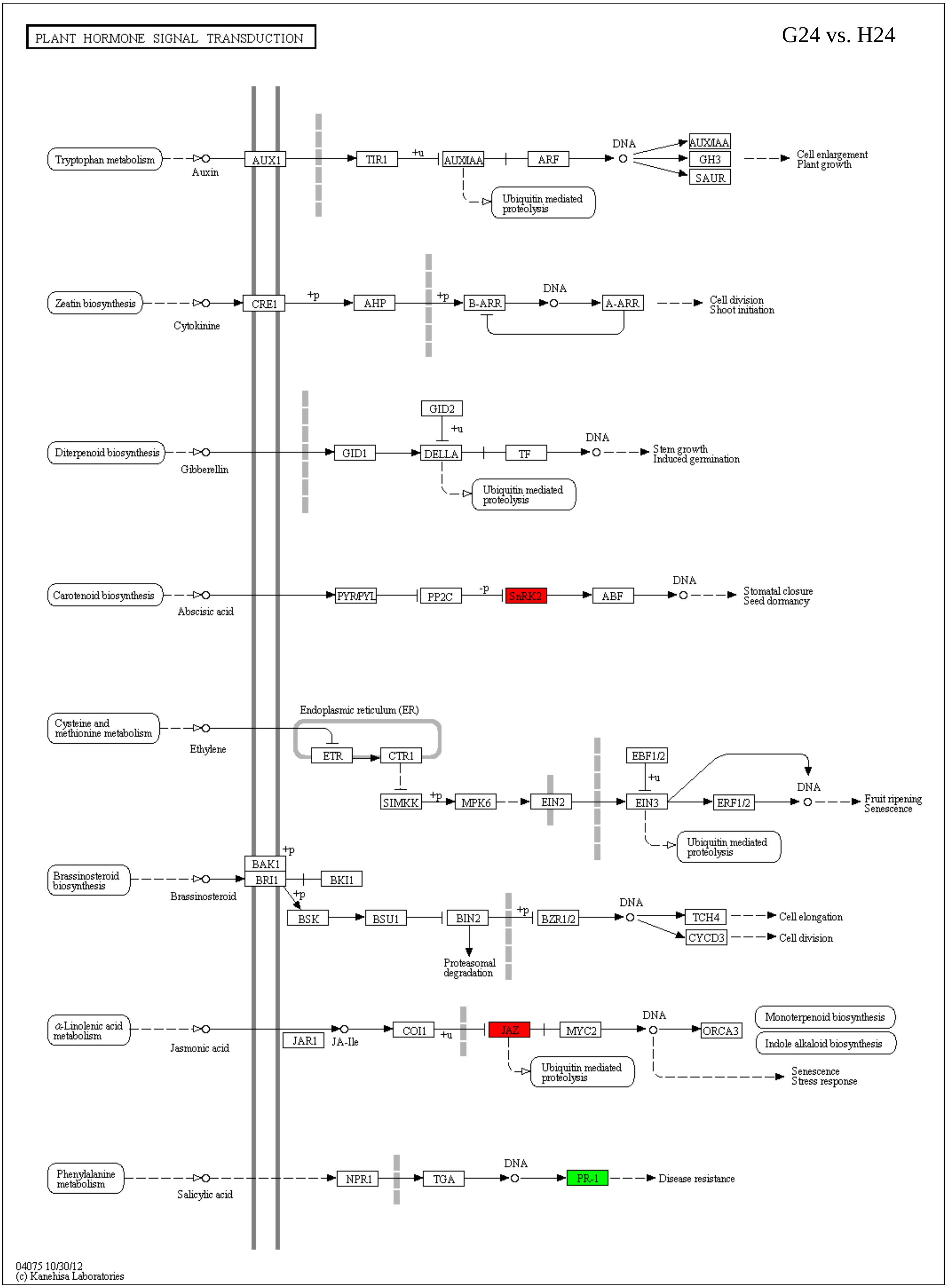

Figure S11 Gene expression in the plant hormone signal transduction pathway (Ko04075) changed in G24 vs. H24 group. The red color indicates gene expression induction in G24 group. The green color indicates gene expression reduction in G24 group.

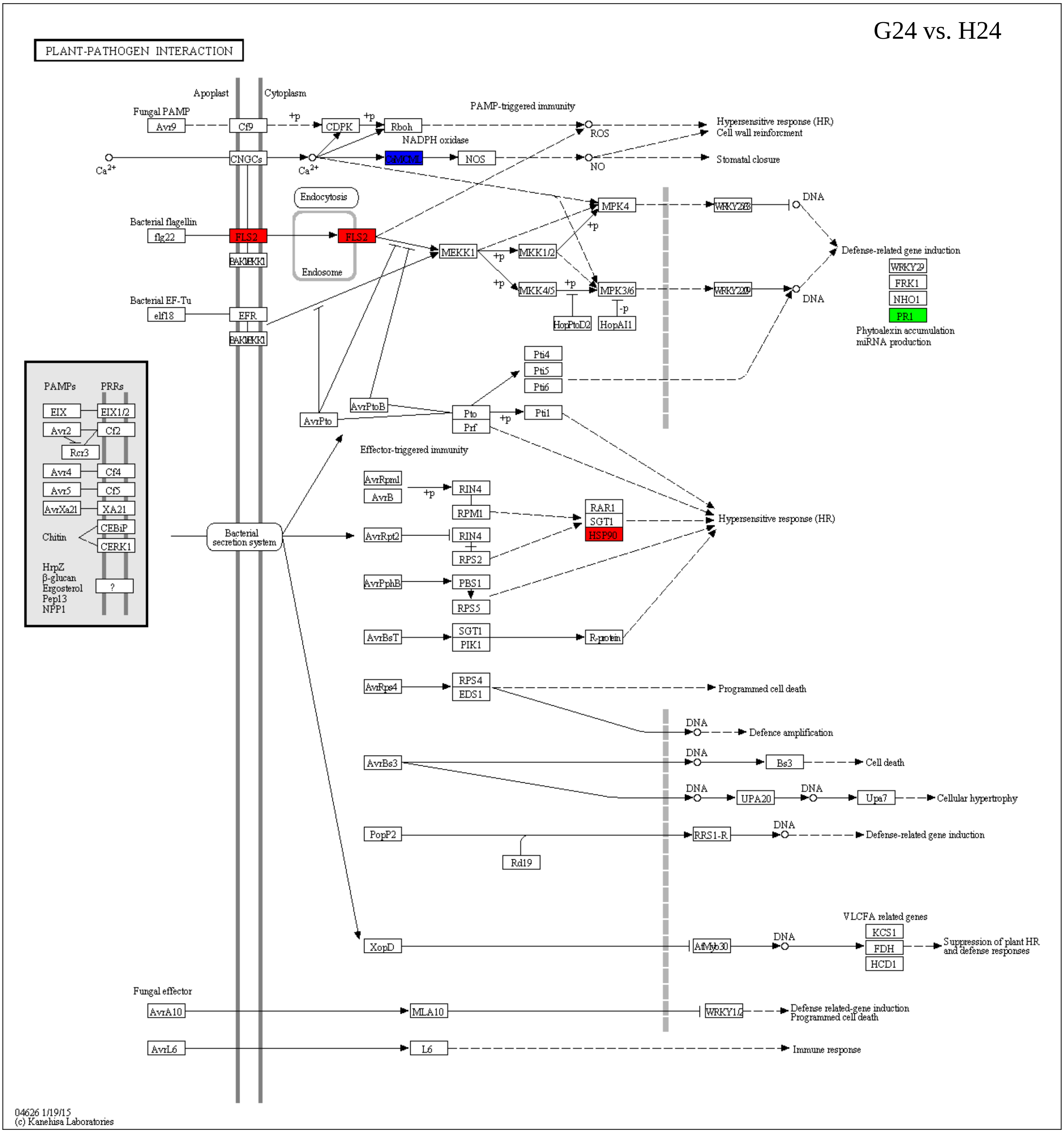

Figure S12 Gene expression in the plant-pathogen interaction pathway (Ko04626) changed in the G24 *vs.* H24 group. The red color indicates gene expression induction in G24 group; The green color indicates gene expression of reduction in G24 group; The blue color indicates that the expression patterns of some annotated genes were induced and some homologous genes were reduced in G24 group.
