# Supplementary material for: Glycerol-Induced Powdery Mildew Resistance in Wheat by Regulating Plant Fatty Acid Metabolism, Plant Hormones Cross-Talk, and Pathogenesis-Related Genes
Source: Int J Mol Sci. 2020 Jan 20;21(2):673. doi: 10.3390/ijms21020673 (PMC7013599; doi:10.3390/ijms21020673)
Supplement: Supplementary file 1 [file ijms-21-00673-s001.zip › supplementary files/Supplement Tables/TableS1.docx]

Table S1: The reads mapped to the wheat genome reference sequence.

| Sample-ID  (BMK-ID) | Total Reads | Mapped Reads | Uniq Mapped Reads | Multiple Map Reads | Reads Map to '+' | Reads Map to '-' |
| --- | --- | --- | --- | --- | --- | --- |
| H0-1  (T01) | 61,381,930 | 53,213,498 (86.69%) | 26,677,479 (43.46%) | 26,536,019 (43.23%) | 16,254,680 (26.48%) | 16,627,507 (27.09%) |
| H0-2  (T02) | 51,779,788 | 44,259,114 (85.48%) | 25,068,802 (48.41%) | 19,190,312 (37.06%) | 14,660,433 (28.31%) | 14,949,726 (28.87%) |
| H0-3  (T03) | 55,555,294 | 46,310,158 (83.36%) | 30,333,984 (54.60%) | 15,976,174 (28.76%) | 16,965,563 (30.54%) | 17,307,670 (31.15%) |
| H24-1  (T04) | 50,710,042 | 41,578,439 (81.99%) | 28,446,532 (56.10%) | 13,131,907 (25.90%) | 15,936,678 (31.43%) | 16,137,485 (31.82%) |
| H24-2  (T05) | 41,214,380 | 34,185,528 (82.95%) | 22,568,921 (54.76%) | 11,616,607 (28.19%) | 12,846,269 (31.17%) | 13,010,846 (31.57%) |
| H24-3  (T06) | 48,928,666 | 40,354,467 (82.48%) | 28,581,424 (58.41%) | 11,773,043 (24.06%) | 15,792,221 (32.28%) | 16,037,970 (32.78%) |
| G0-1  (T07) | 66,987,444 | 55,484,395 (82.83%) | 40,169,879 (59.97%) | 15,314,516 (22.86%) | 22,072,726 (32.95%) | 22,399,423 (33.44%) |
| G0-2  (T08) | 47,942,810 | 39,928,146 (83.28%) | 28,305,647 (59.04%) | 11,622,499 (24.24%) | 15,603,910 (32.55%) | 15,821,643 (33.00%) |
| G0-3  (T09) | 51,286,994 | 43,415,159 (84.65%) | 24,254,787 (47.29%) | 19,160,372 (37.36%) | 14,384,173 (28.05%) | 14,671,353 (28.61%) |
| G24-1  (T10) | 68,266,986 | 56,452,182 (82.69%) | 38,436,731 (56.30%) | 18,015,451 (26.39%) | 21,581,789 (31.61%) | 21,856,863 (32.02%) |
| G24-2  (T11) | 59,663,806 | 49,420,379 (82.83%) | 31,866,922 (53.41%) | 17,553,457 (29.42%) | 18,339,503 (30.74%) | 18,654,390 (31.27%) |
| G24-3  (T12) | 77,987,478 | 64,189,560 (82.31%) | 44,428,809 (56.97%) | 19,760,751 (25.34%) | 24,764,042 (31.75%) | 25,104,093 (32.19%) |

Note: ‘+’indicates positive-sense strand of DNA; ‘-’ indicates antisense strand of DNA.
