# Supplementary material for: Glycerol-Induced Powdery Mildew Resistance in Wheat by Regulating Plant Fatty Acid Metabolism, Plant Hormones Cross-Talk, and Pathogenesis-Related Genes
Source: Int J Mol Sci. 2020 Jan 20;21(2):673. doi: 10.3390/ijms21020673 (PMC7013599; doi:10.3390/ijms21020673)
Supplement: Supplementary file 1 [file ijms-21-00673-s001.zip › supplementary files/Supplement Tables/TableS10.docx]

Table S10: DEGs in the G24 vs. H24 group.

|  | #ID | H0 | G0 | **H24** | **G24** | Annotation |
| --- | --- | --- | --- | --- | --- | --- |
| *1.13.11.12* | Traes_5DS_A281EFAB4 | 0.50 | 0.52 | **0.10** | **0.57** | Lipoxygenase 2.2, chloroplastic |
| *1.13.11.58* | Traes_4BS_63DD9D036 | 2.13 | 5.62 | **5.75** | **10.62** | Linoleate 9S-lipoxygenase 1 |
|  | Traes_4BS_71CB57A0D | 4.33 | 15.90 | **8.60** | **17.88** |  |
|  | Traes_4DS_7868A8C2E | 2.13 | 11.58 | **7.16** | **18.30** |  |
|  | Triticum_aestivumLinn_newGene_11732 | 1.44 | 13.99 | **0.68** | **17.84** | Putative linoleate 9S-lipoxygenase 3 PE=3 SV=1 |
| *4.2.1.92* | Traes_4AS_41FB87D39 | 2.37 | 21.67 | **5.56** | **24.73** | Allene oxide synthase 2 |
|  | Traes_4AS_9F1B2A7DD | 1.66 | 14.56 | **1.00** | **7.24** |  |
|  | Traes_4BL_523D155E21 | 2.63 | 43.79 | **6.79** | **49.98** |  |
|  | Traes_4BL_C01E043B6 | 1.10 | 10.83 | **1.46** | **7.70** |  |
|  | Traes_4BL_DD6DD7487 | 17.33 | 119.91 | **38.96** | **170.11** |  |
|  | Traes_4BL_EC6D20026 | 5.55 | 115.08 | **29.68** | **122.97** |  |
|  | Traes_4DL_B3E978E9F | 1.76 | 14.96 | **1.02** | **6.22** |  |
|  | Traes_5BL_E13A2FBD01 | 1.13 | 1.75 | **1.80** | **3.23** |  |
| *5.3.99.6* | Traes_6AL_C11B22ED8 | 2.84 | 8.73 | **2.98** | **10.36** | allene oxide cyclase |
| *FLS2* | Traes_2BL_6E8764762 | 0.21 | 0.39 | **0.37** | **0.68** | LRR receptor-like serine/threonine-protein kinase FLS2 (Precursor) |
| *HSP90* | Traes_2AS_67EFE0FAE | 0.24 | 1.53 | **0.29** | **0.79** | heat shock protein 90 |
|  | Traes_2DS_3B16D8173 | 0.09 | 1.58 | **0.42** | **0.83** |  |
| *JAZ* | Traes_5BL_7A6C3831E | 0.36 | 0.58 | **0.31** | **0.97** | Protein TIFY 10B |
|  | Traes_5DL_4186C5347 | 0.34 | 1.60 | **0.40** | **1.46** | Protein TIFY 10A |
| *SnPK2* | Traes_2AL_2FF604DA9 | 0.35 | 0.89 | **0.35** | **1.20** | Abscisic acid-inducible protein kinase |
| *Stress-related genes* | Traes_5BL_62D9B877B | 3.86 | 8.54 | **4.97** | **10.35** | Aldehyde dehydrogenase  family 7 |
|  | Traes_5DL_A2A5A19B4 | 1.41 | 8.97 | **2.56** | **11.23** |  |
|  | Traes_2AL_6A8D574C4 | 0.32 | 3.42 | **0.25** | **2.85** | Peroxygenase |
|  | Traes_5DL_EEF38A7E4 | 0.23 | 3.51 | **6.41** | **13.68** | Chitinase 8 |
|  | Traes_2AL_608FCBC83 | 0.42 | 1.98 | **2.31** | **5.51** | D-3-phosphoglycerate dehydrogenase 1, chloroplastic |
|  | Traes_7DL_A2604A6D5 | 0.96 | 6.92 | **5.87** | **16.09** | putative gibberellin 20-oxidase |
|  | Traes_1AL_326B4C863 | 2.16 | 7.18 | **8.25** | **27.37** | Bowman-Birk type wound-induced proteinase inhibitor WIP1 (Precursor) |
|  | Traes_3DL_B38DFDDFF | 0.39 | 11.67 | **2.54** | **22.57** | Peroxidase 2 |
|  | Traes_5BL_36EBD512B | 0.20 | 0.94 | **0.77** | **1.91** | Peroxidase 35 |
|  | Traes_4AL_F4D7A0D14 | 0.24 | 2.15 | **0.37** | **1.37** | Phenolic glucoside malonyltransferase 1 |
|  | Traes_4DS_1D6698102 | 1.04 | 2.32 | **1.10** | **2.77** |  |
|  | Traes_4DS_51D4A19F4 | 0.66 | 3.69 | **1.34** | **3.29** |  |
|  | Traes_6BS_539C8C254 | 0.09 | 5.87 | **2.01** | **9.72** | Subtilisin-chymotrypsin inhibitor-2A |
|  | Traes_1BS_FCCC2FA60 | 0.12 | 2.92 | **0.09** | **1.69** |  |
|  | Traes_3AS_075D3EB21 | 0.86 | 4.97 | **2.22** | **6.05** |  |
|  | TRAES3BF012400020CFD_g | 0.43 | 2.17 | **0.80** | **2.82** | xylanase inhibitor 801NEW |
|  | Traes_6AS_8ED966774 | 0.34 | 2.49 | **1.38** | **6.42** | xylanase inhibitor XIP-III |
|  | Traes_1BL_A6F7A9A54 | 0.60 | 1.39 | **0.45** | **1.03** | Glucan endo-1,3-beta-glucosidase 14 (Precursor) |
|  | Traes_2BS_8B4CB340F | 0.85 | 1.37 | **0.43** | **1.35** | Beta-glucosidase 16 |
|  | Traes_5DL_DEAD02939 | 0.87 | 2.15 | **0.72** | **2.41** | UDP-glycosyltransferase 83A1 |
|  | Traes_5BL_7F59B65A3 | 1.03 | 6.65 | **2.77** | **6.83** | UDP-glucose 6-dehydrogenase 4 |
|  | Traes_5DL_0A7630D1E | 2.61 | 18.93 | **7.58** | **19.82** |  |
