# Supplementary material for: Glycerol-Induced Powdery Mildew Resistance in Wheat by Regulating Plant Fatty Acid Metabolism, Plant Hormones Cross-Talk, and Pathogenesis-Related Genes
Source: Int J Mol Sci. 2020 Jan 20;21(2):673. doi: 10.3390/ijms21020673 (PMC7013599; doi:10.3390/ijms21020673)
Supplement: Supplementary file 1 [file ijms-21-00673-s001.zip › supplementary files/Supplement Tables/TableS2.docx]

Table S2: The number of annotated genes identified in searches of six databases.

| #Anno_Database | Annotated_Number | 300<=length<1000 | length>=1000 |
| --- | --- | --- | --- |
| COG_Annotation | 36839 | 16729 | 15757 |
| GO_Annotation | 90938 | 40326 | 31849 |
| KEGG_Annotation | 38112 | 17331 | 13442 |
| KOG_Annotation | 55561 | 24928 | 20742 |
| Swissprot_Annotation | 77973 | 34566 | 29717 |
| nr_Annotation | 106702 | 46843 | 37550 |
| All_Annotated | 106755 | 46865 | 37571 |
