# Supplementary material for: Glycerol-Induced Powdery Mildew Resistance in Wheat by Regulating Plant Fatty Acid Metabolism, Plant Hormones Cross-Talk, and Pathogenesis-Related Genes
Source: Int J Mol Sci. 2020 Jan 20;21(2):673. doi: 10.3390/ijms21020673 (PMC7013599; doi:10.3390/ijms21020673)
Supplement: Supplementary file 1 [file ijms-21-00673-s001.zip › supplementary files/Supplement Tables/TableS4.docx]

Table S4: The numbers of DEGs in different GO functional categorization terms.

| GO_classify | All Unigene | DEGs  (H0 vs. H24) | DEG s  (G0 vs. 24) | DEG s  (H0 vs. G0) | DEG s  (G24 vs. H24) |
| --- | --- | --- | --- | --- | --- |
| Total_gene | 90939 | 4938 | 2206 | 382 | 412 |
| **cellular component** |  |  |  |  |  |
| extracellular region | 8827 | 651 | 369 | 107 | 109 |
| collagen trimer | 3 | 0 | 0 | 0 | 0 |
| cell | 78040 | 4375 | 1928 | 323 | 356 |
| nucleoid | 285 | 3 | 0 | 0 | 0 |
| membrane | 40000 | 2535 | 1137 | 166 | 186 |
| virion | 23 | 3 | 0 | 0 | 0 |
| cell junction | 9049 | 630 | 308 | 60 | 54 |
| extracellular matrix | 256 | 12 | 4 | 0 | 4 |
| membrane-enclosed lumen | 2813 | 157 | 61 | 12 | 2 |
| macromolecular complex | 14045 | 808 | 266 | 36 | 22 |
| organelle | 71770 | 4032 | 1771 | 301 | 329 |
| extracellular matrix part | 50 | 4 | 3 | 0 | 1 |
| extracellular region part | 374 | 29 | 17 | 4 | 5 |
| organelle part | 27716 | 1770 | 715 | 115 | 110 |
| virion part | 23 | 3 | 0 | 0 | 0 |
| membrane part | 15476 | 906 | 404 | 63 | 61 |
| cell part | 79227 | 4428 | 1950 | 325 | 359 |
| protein binding transcription factor activity | 385 | 16 | 5 | 2 | 0 |
| nucleic acid binding transcription factor activity | 4337 | 207 | 125 | 13 | 12 |
| **molecular function** |  |  |  |  |  |
| catalytic activity | 48284 | 2939 | 1358 | 276 | 300 |
| receptor activity | 1584 | 104 | 64 | 3 | 7 |
| guanyl-nucleotide exchange factor activity | 114 | 11 | 0 | 0 | 0 |
| structural molecule activity | 2918 | 271 | 112 | 1 | 2 |
| transporter activity | 7233 | 410 | 195 | 25 | 24 |
| binding | 54179 | 2955 | 1333 | 222 | 260 |
| electron carrier activity | 2884 | 164 | 85 | 19 | 20 |
| antioxidant activity | 1083 | 93 | 62 | 13 | 27 |
| channel regulator activity | 9 | 1 | 0 | 0 | 0 |
| metallochaperone activity | 34 | 1 | 0 | 0 | 0 |
| enzyme regulator activity | 1171 | 62 | 31 | 16 | 10 |
| protein tag | 10 | 0 | 0 | 0 | 0 |
| translation regulator activity | 27 | 3 | 1 | 0 | 0 |
| nutrient reservoir activity | 428 | 40 | 20 | 2 | 9 |
| molecular transducer activity | 2324 | 133 | 81 | 0 | 8 |
| **biological process** |  |  |  |  |  |
| reproduction | 5583 | 261 | 108 | 18 | 19 |
| cell killing | 84 | 16 | 14 | 3 | 3 |
| immune system process | 7970 | 537 | 312 | 48 | 41 |
| metabolic process | 66839 | 4025 | 1824 | 324 | 352 |
| cellular process | 68286 | 4017 | 1818 | 310 | 335 |
| reproductive process | 18984 | 999 | 482 | 76 | 86 |
| biological adhesion | 775 | 38 | 12 | 2 | 2 |
| signaling | 13129 | 765 | 441 | 61 | 75 |
| multicellular organismal process | 26020 | 1472 | 670 | 121 | 126 |
| developmental process | 31504 | 1786 | 830 | 150 | 165 |
| growth | 9790 | 491 | 237 | 48 | 49 |
| locomotion | 225 | 3 | 3 | 0 | 1 |
| single-organism process | 62038 | 3679 | 1641 | 296 | 326 |
| biological phase | 296 | 15 | 6 | 0 | 0 |
| rhythmic process | 1095 | 45 | 19 | 6 | 4 |
| response to stimulus | 45833 | 2931 | 1445 | 298 | 320 |
| localization | 26801 | 1651 | 822 | 139 | 143 |
| multi-organism process | 17227 | 1183 | 694 | 120 | 134 |
| biological regulation | 38438 | 2163 | 1015 | 183 | 191 |
| cellular component organization or biogenesis | 31731 | 1875 | 828 | 142 | 139 |
