# Supplementary material for: Glycerol-Induced Powdery Mildew Resistance in Wheat by Regulating Plant Fatty Acid Metabolism, Plant Hormones Cross-Talk, and Pathogenesis-Related Genes
Source: Int J Mol Sci. 2020 Jan 20;21(2):673. doi: 10.3390/ijms21020673 (PMC7013599; doi:10.3390/ijms21020673)
Supplement: Supplementary file 1 [file ijms-21-00673-s001.zip › supplementary files/Supplement Tables/TableS6.docx]

|  | #ID | **H0** | **H24** | G0 | G24 | nr_annotation |
| --- | --- | --- | --- | --- | --- | --- |
| *2.7.1.30*  *(TaGLI1)* | Traes_2AL_B418D6172 | **9.10** | **21.75** | 8.87 | 17.01 | Glycerol kinase |
|  | Traes_2BL_739A3AD20 | **6.90** | **11.43** | 8.10 | 11.38 |  |
|  | Traes_2DL_ABA700EBD | **9.62** | **18.63** | 10.28 | 18.31 |  |
| *1.1.1.21* | Traes_1BL_4D2CB33FC | **0.45** | **1.82** | 1.93 | 3.67 | Aldo-keto reductase family 4 member C10 |
| *2.3.1.15*  *(TaACT1)* | Triticum_aestivumLinn_newGene_5098 | **1.39** | **0.73** | 1.15 | 0.85 | Glycerol-3-phosphate acyltransferase 1 |
| *1.14.192*  *(TaSSI2)* | Traes_2AL_D4BF4AE24 | **2.43** | **3.33** | 2.75 | 3.58 | Acyl-[acyl-carrier-protein] desaturase 5 |
|  | Traes_2BL_11A4F903B | **0.93** | **1.86** | 1.19 | 1.96 |  |
|  | Traes_2DL_8F04980F0 | **4.71** | **9.42** | 6.38 | 9.55 |  |
| *FabI* | Traes_7AL_3A910D814 | **0.69** | **1.56** | 0.83 | 1.19 | Enoyl-[acyl-carrier-protein] reductase [NADH] 1, chloroplastic (Precursor) |
| *FabZ* | Traes_2AL_A711DF57E1 | **0.19** | **1.20** | 0.59 | 1.10 | 3-hydroxyacyl-[acyl-carrier-protein] dehydratase |
| *FabF* | Traes_7DS_AD88E0A2F | **0.27** | **0.74** | 0.48 | 0.69 | 3-oxoacyl-(acyl-carrier-protein) synthase I, chloroplastic |
| *FabD* | Traes_4BL_CB4933F58 | **0.48** | **1.61** | 0.92 | 1.34 | Malonyl-CoA-acyl carrier protein transacylase, mitochondrial |
|  | Traes_4DL_9BBFDE200 | **0.88** | **2.09** | 1.09 | 1.39 |  |

Table S6: DEGs involved in glycerolipid metabolism and fatty acid biosynthesis pathways in response to *Bgt* infection (H24 vs. H0).
